# Estimating the Burden of Cost in Chronic Graft-versus-Host Disease: A Human Capital Approach

**DOI:** 10.36469/9814

**Published:** 2016-06-01

**Authors:** Chris A. Jones, Luca P. Fernandez, Peter Weimersheimer, Neil A. Zakai, Michael Sharf, Oscar A. Mesa, Christian Peters, Antonio di Carlo, Mitchell C. Norotsky

**Affiliations:** 1 Department of Surgery Global Health Economics Unit of the Vermont Center for Clinical and Translational Science, Burlington, VT (USA); University of Vermont College of Medicine, Burlington, VT (USA); 2 Global Health Economics Unit of the Vermont Center for Clinical and Translational Science, Burlington, VT (USA); 3 Department of Surgery; Division of Emergency Medicine University of Vermont College of Medicine, Burlington, VT; University of Vermont Medical Center, Burlington, VT (USA); 4 Department of Medicine University of Vermont, Burlington, VT (USA); 5 Therakos, Inc., West Chester, PA (USA); 6 Division of Abdominal Organ Transplant Program Department of Surgery, Temple University School of Medicine, Philadelphia, PA (USA); 7 Department of Surgery University of Vermont College of Medicine, Burlington, VT (USA)

**Keywords:** lost work time, societal perspective, health economics, extracorporeal photopheresis, transplant, graft versus host disease, stem cell transplant, chronic gvhd

## Abstract

With advances in organ matching and preventing acute graft-versus-host-disease (aGvHD), chronic graft-versus-host disease (cGvHD) following allogeneic hematopoietic stem cell transplantation (HSCT) has become a focus of transplant-related morbidity and mortality. Given that cGvHD often presents years following a transplant, our objective was to estimate its burden of cost resulting from allogeneic HSCT based on published estimates of incidence, morbidity, the value of lost work time and survivorship. Our choice of a ten-year time horizon is novel to the field of rare disease and was determined to be meaningful after consultations with present co-authors, including five physicians, one of whom is a transplant surgeon. A total of 44 450 cGvHD patients in the United States were estimated to require treatment over the next decade (from 2015 to 2025). This estimate is based on the last 5 years of trends reported in the transplant registries. What is not reported in any registry is that these patients will accrue a total of 605 631 years of lost wages, a collective lost productivity that will cost society over $27 Billion in the decade ahead: more than five times ($27B vs. $5.2B) the estimated ten-year cost of treating the condition.

## INTRODUCTION

Of all the present-day solid organs or tissues that are transplanted, hematopoietic (i.e. blood-producing) stem cells and lungs are associated with much higher rates of complication and rejection, both immediately following surgery and throughout the patient's life. When introducing foreign tissue such as HSCs, the patient's own immune system that has evolved to reject foreign antigens is essentially replaced with a new immune system. Consequent sequelae are from the graft identifying the patient's own body as foreign.

Autologous Stem Cell Transplantation (ASCT) uses the patient's own cells, and therefore rarely results in complications. Whereas this procedure carries less risk for the patient, it also does not offer the added benefit of the "graft-versus-disease (GVD) effect", resulting in less net immunocompetency to the host.[1] A graft transplanted from a compatible but "non-self" donor can attack disease cells which the host's weakened immune system is unable to do. As such, 40% of stem cell transplant recipients receive the riskier but potentially much more beneficial allogeneic stem cell transplantation. The described risk of complication is significant, however. Allogeneic stem cell transplantation can require high doses of chemotherapy or radiation therapy in order to prepare a recipient for the procedure. This therapy weakens the hosts' immune systems along with its ability to deliver healthy, potent stem cells.[1]

While the benefits of graft with respect to disease may pay off for many patients, the downstream risks of cGvHD remain significant. The GvHD mortality rate for GVD is approximately 25%; graft rejection will occur in 50% of patients. 50% of patients who survive will have significant morbidity, often suffering from long periods of illness. From the wage earning or human capital perspective, 25% of survivors will be never be able to return to paid employment.[2]

Economics is concerned with finite resources and efficiency. The specialty of health economics adopts the societal perspective wherever possible. Adopting this wider perspective, the following analyses demonstrate that whereas the medical costs associated with treating cGvHD are lifelong, the total medical burden of illness may be overshadowed by the much higher cost to society of lost productivity from cGvHD patients who are unable, as a result of their condition, to return to paid employment.

## METHODS

Prevalence, incidence, morbidity and mortality estimates were obtained from published sources including the Bone Marrow Transplant Survivor Study (BMTSS). Treatment pathways and adverse events were evaluated in terms of direct cost from published primary and secondary sources. Additional cost estimates for readmission and follow-up care were annualized and compared between non-cGvHD patients and differential levels of cGvHD severity over a 5-year period, based on published studies conducted in the United States and United Kingdom. Indirect costs (or benefits) were calculated based on age-adjusted United States Census Bureau reported average wages, wage growth and the probability that ability to work would be impaired or foregone. Currency was adjusted to 2014 USD. Extreme scenario analyses were used to assess the robustness of the model.

Within a broad interpretation of the value of a statistical life, we estimated a societal burden of cost resulting from a conservative combination of lost human capital and added medical expenditures. From this wider societal perspective, total cost surrounding GvHD over the next decade was evaluated based on the formula: Total Cost (TC) = Prevalent Population (PP) * (Medical Cost [MC] + Lost Wages [LWT]) * time (t in years).

The data populating this formula were derived from several population-based databases and secondary sources, as thusly described. Transplant statistics were gathered using data from the Leukemia and Lymphoma Society.[1] Prevalence of cGvHD, time delay to onset treatment costs, and average wage assumptions for the United States were summated and projected over a ten-year time horizon for the predicted cGvHD population in 2025.[1,3] Medical costs were adopted from Crespo et al. (2012), who provided a 5-year cumulative cost for treating cGvHD patients with immunosuppressants alongside the cost of extracorporeal photopheresis.[4] Inflationary adjustments were applied based on indices reported by the Bureau of Labor Statistics.[5]

## ANALYSIS

A key assumption in this analysis is the employment rate prior to transplant. Often, rates of employment for transplant patients are low because of the impact of the illness on cognitive and physical functioning. As prior employment rates have not been published for HTC patients, we applied sensitivity analysis to a range of employment rates. Our baseline calculations assume full employment. We additionally calculated the human capital impact for a range {75%, 25%} of pre-illness employment estimates that were specific to our cGvHD population.

Prevalence estimates were derived from the Leukemia and Lymphoma Society's report "Blood Marrow and Stem Cell Transplantation."[1] The LLS reported 7000 transplants in North America during 2013. With a 50% incidence rate of cGvHD amongst allogeneic transplants, this creates 3500 cGvHD patients each year, with a 45-50% survival rate at five years.[6]

Of note, even a patient with a short prognosis will accrue most of the costs associated with cGvHD.[4] Whereas 75% of the surviving patients are expected to fully recover and only lose three years of earnings, the remaining 25% will each lose 20 years of earnings due to permanent disability. When evaluated in this light, only 37.5 percent of cGvHD patients, on the average, will ever be able to return to the workforce. A total of 44 450 cGvHD patients were estimated from the last 5 years of trends reported in the registries, to require treatment over the next decade (from 2015 to 2025). These patients will present 605 631 years of lost wages ((44 450*0.5*0.75)*3)+(44 450*0.5*0.25*20)+(44 450*0.5*20).

Typically, cGvHD patients will receive topical steroids, topical FK506 and other agents such as leukotriene inhibitors and azithromycin, all of which present cumulative costs. Crespo et al. (2012) reported a 5-year cumulative medical cost of $118 629.47 for treating cGvHD.[4] Applying this to our 9450 prevalent chronic GvHD patients with an additional 3,500 incident patients a year for ten years yield $5273 079 941.50 for treating our population. Adding to this the $27 185 672 451 in lost wages that accrue from 605 631.25 lost years at an average income of $44 888.16 as reported by the Social Security Administration. With a total 10-year burden of cost that exceeds $32B, side by side comparison shows the lost work time component will cost more than 5X ($27B vs. $5.2B) the estimated ten-year cost of treating cGvHD, including diagnostics, immunosuppressives, and additional complications.

**Table 1.** Ten Year Burden of Cost for cGvHD

| | |
|---|---|
| Estimated Prevalence | 9450 |
| Estimated Incidence | 3500 |
| 10-year Incidence | 35 000 |
| Total | 44 450 |
| Lost Years of Wages | 605 631 |
| Lost Wages | $27 185 672 451 |
| 10-year Medical Cost | $5273 079 941 |
| Total 10-year Cost | $32 458 752 392 |

**Table 2.** Sensitivity Analysis

| Analysis | Years Lost | Wages Lost | Total W/Medical Cost | % Baseline |
|---|---|---|---|---|
| **Baseline** | | | | |
| | 605 631 | $27 185 672 451.00 | $32 458 752 392.00 | 1 |
| **Percent Employed Prior to cGvHD Diagnosis** | | | | |
| 0.75 | 454 223 | $20 389 254 338.25 | $25 662 334 279.75 | 0.79 |
| 0.50 | 302 816 | $13 592 836 225.50 | $18 865 916 167.00 | 0.58 |
| 0.25 | 151 408 | $6 796 418 112.75 | $12 069 498 054.25 | 0.37 |
| **Percent Survivors Disabled** | | | | |
| 0.20 | 586 740 | $26 337 678 998.40 | $31 610 758 939.90 | 0.97 |
| 0.30 | 624 522 | $28 033 665 903.60 | $33 306 745 845.10 | 1.02 |
| 0.40 | 662 305 | $29 729 652 808.80 | $35 002 732 750.30 | 1.07 |
| 0.50 | 700 087 | $31 425 639 714.00 | $36 698 719 655.50 | 1.13 |
| **Years Lost to Mortality** | | | | |
| 15 | 466 725 | $20 950 426 476.00 | $26 223 506 417.50 | 0.81 |
| 25 | 744 537 | $33 420 918 426.00 | $38 693 998 367.50 | 1.19 |
| 30 | 883 443.75 | $39 656 164 401.00 | $44 929 244 342.50 | 1.38 |
| 40 | 1161 256.25 | $52 126 656 351.00 | $57 399 736 292.50 | 1.77 |
| **Survivors- Duration of Illness** | | | | |
| 1 | 572 293.75 | $25 689 213 417.00 | $30 962 293 358.50 | 0.95 |
| 2 | 588 962 | $26 437 442 934.00 | $31 710 522 875.50 | 0.98 |
| 4 | 622 300 | $27 933 901 968.00 | $33 206 981 909.50 | 1.02 |
| 5 | 638 968.75 | $28 682 131 485.00 | $33 955 211 426.50 | 1.05 |

## DISCUSSION

From the societal perspective, the human capital lost due to cGvHD will cost society many times more than the cost of treating the condition. As such, GvhD is estimated to present a burden of cost that will exceed $27 Billion over the next decade in the United States alone. The sensitivity analysis shows that the average age of the patient, which is reflected in the number of work years lost due to mortality, has the highest effect on outcomes. The $27 Billion (human capital) forfeiture is age-dependent; transplants in younger populations create a higher societal cost. A decade of change in the average years lost results in a 38% change in total burden of cost—without changing the medical cost.

Inhibiting performance of Allogeneic HCST in unlikely long-term survival cases may significantly reduce costs. It is well known that much of an individual's health care expenses occur during their last months of life. The $5.2 Billion substantial treatment cost accounts for 16% of the 10-year total and includes many operations in elderly patients with co-morbidities and minimal chance of endurance. Adopting efficient practices that are mindful of quality of life in this population will reduce medical cost and may be warranted due to the high risk of HCST, resulting GvhD, and the improbability of long-term survival. The future of reduced intensity allogenic transplants may also lead to an increased prevalence of chronic GvHD for patients who would otherwise have experienced acute rejection.

The total burden of cost from cGvHD has traditionally been calculated based on the cost of transplant and normative follow-up. However, this cost is far more poignant when viewing long-term morbidity, mortality, and consequent lost wages. Relapse due to primary disease (29%) and cGvHD (22%) were reported by the literature to be the leading causes of premature mortality. This is important in the counter-factual scenario where patients might have returned to normal daily activities. Taking one example in a per-patient estimate, the mean age for developing cGvHD was modeled from 51 years for a person earning $50 000, per year, with an estimated span of 14 years of potential employment prior to retirement. This person alone in being prevented from working would yield a human capital foregone of $700 000. Such a figure exceeds the reported hospital costs of even complicated cases. Including caregiver costs as Majhail and colleagues have estimated (2013)7, would intensify the medical, family and societal burden of this rare disease.

This figure also exceeds our earlier calculations[8] for the burden of cost in graft versus host disease as well as recent estimates[9] for the burden of cost in bronchiolitis obliterans syndrome. With the total burden of cost for cGvHD presented as a summation of direct and indirect components, our results show the profound manner by which cumulative lost worktime will grow over just one decade. With the exception of sickle cell disease, the diseases treated by allogeneic transplant appear to strike at random without demographic or socioeconomic markers from which to predict disease or outcomes. Transplant eligibility, however, requires a great deal of family and social support. Including this broader perspective of additional lost work time from family and caregivers would only intensify our ten-year findings.

## CONCLUSIONS

In the current state of rising healthcare costs, treatments for chronic conditions should be modeled and evaluated over a ten year time period. The ability to return to daily functioning, from a human capital approach, cannot be separated from other cost-effectiveness denominators. In the case of cGvHD that is secondary to HSCT, health technology assessment should include a wider societal perspective that includes modeling the ability or inability to return to paid employment.

## DISCLAIMER

Financial: ForMyOdds LLC, a company of which CJ is principal, has consulted to Therakos Inc. on design of this and other health economic studies.

Reputational: CJ and CP were colleagues in a publicly-traded corporation working in areas unrelated to the topic of this paper.

Travel: None.
